# Tackling Antibiotic Resistance: Exploring 5-Fluorouracil as a Promising Antimicrobial Strategy for the Treatment of *Streptococcus suis* Infection

**DOI:** 10.3390/ani14091286

**Published:** 2024-04-24

**Authors:** Jing Zuo, Yingying Quan, Jinpeng Li, Yue Li, Dong Song, Xingping Li, Yuxin Wang, Li Yi, Yang Wang

**Affiliations:** 1 College of Animal Science and Technology, Henan University of Science and Technology, Luoyang 471000, China; z4379479232021@163.com (J.Z.); quan00826@163.com (Y.Q.); 18739929053@163.com (Y.L.); songdongss@163.com (D.S.); lxp@haust.edu.cn (X.L.); wangyuxin_1991@163.com (Y.W.); 2 Henan Provincial Engineering Research Center for Detection and Prevention and Control of Emerging Infectious Diseases in Livestock and Poultry, Luoyang 471003, China; jinpeng5070@163.com; 3 Animal Disease Prevention and Food Safety Key Laboratory of Sichuan Province, College of Life Sciences, Sichuan University, Chengdu 610064, China; 4 College of Life Science, Luoyang Normal University, Luoyang 471934, China

**Keywords:** *Streptococcus suis*, 5-fluorouracil, antimicrobial agent, antibiotic resistance, thymine-less death

## Abstract

**Simple Summary:**

The importance of exploring new antibacterial alternatives to combat *Streptococcus suis* (*S. suis*) infections cannot be overstated. While the application of 5-fluorouracil (5-FU) to other bacteria has demonstrated initial success, its antibacterial effect in *S. suis* remains largely unexplored. In this study, we demonstrated that various pathogens, especially *S. suis*, are more sensitive to 5-FU. Moreover, the cytotoxicity of 5-FU is relatively low. Additionally, we preliminarily determined that 5-FU mainly acts on *S. suis* by destroying bacterial cell walls and membranes, leading to the leakage of intracellular components, as well as inhibiting thymidine synthesis, leading to thymine-less death and lethal DNA damage in bacteria. We show that 5-FU dramatically attenuates a murine infection. These results highlight the antimicrobial potential of 5-FU against *S. suis* and provide evidence for its ability to target bacterial membrane damage and DNA damage. This suggests that 5-FU can effectively control *S. suis* infection and may emerge as a promising alternative to antibiotics.

**Abstract:**

*Streptococcus suis* (*S. suis*) is a zoonotic pathogen with a global distribution, which causes serious diseases in both humans and animals and economic losses in the swine industry. As antibiotic resistance increases, there is an urgent imperative to explore novel antibacterial alternatives. In the present study, we selected the anticancer drug 5-fluorouracil (5-FU) approved by the Food and Drug Administration (FDA) as a candidate drug to treat *S. suis* infections. The results showed that various pathogens, especially *S. suis*, are more sensitive to 5-FU. Moreover, the cytotoxicity of 5-FU is relatively low. Extensive in vitro assays demonstrated the pronounced bacteriostatic and bactericidal efficacy of 5-FU against susceptible and multidrug-resistant *S. suis* strains. Its mechanisms of action include damage to the bacterial cell walls and membranes, resulting in the leakage of intracellular components, and the inhibition of thymidylate synthase (TS), leading to a depletion of deoxythymidine triphosphate (dTTP) pools, ultimately causing thymine-less death and lethal DNA damage in bacteria. Gene-knockout experiments further showed that 5-FU played a role by inhibiting the *thyA* gene-encoding thymidine synthase. Finally, we determined that *S. suis* infections can be alleviated by 5-FU in the mouse infection model. This study emphasizes the antibacterial potential of 5-FU against *S. suis* and provides evidence for its targeting of bacterial membrane damage and DNA damage. In summary, 5-FU can control *S. suis* infection and is expected to become a new alternative to antibiotics.

## 1. Introduction

*Streptococcus suis* (*S. suis*) is an important zoonotic pathogen widely distributed throughout the world [1,2], which can cause a variety of diseases in humans and animals. For example, it can cause acute septicemia, pneumonia, meningitis, endocarditis, arthritis, and other diseases in pigs, as well as bacterial meningitis, streptococcal toxic shock syndrome, and other diseases in humans [3]. In 1998 and 2005, there were outbreaks of *S. suis* type-2 infections in Jiangsu and Sichuan, with hundreds of cases and dozens of deaths [1,4]. In recent years, due to the extensive and irrational use of antibiotics, the drug resistance of *S. suis* has gradually increased [5], which makes the infection prevention and control of *S. suis* more difficult. *S. suis* infections cause huge economic losses to the livestock industry every year, seriously affecting the development of the swine industry [6]. Because of the implementation of policies related to the reduction or elimination of the use of antibiotics, *S. suis* infection faces a dilemma in which no drug is available, so there is an urgent need to find new antibacterial drugs. Due to the difficulty in identifying new chemical antibiotics, the development speed of new antibiotics is not keeping pace with increasing resistance [7]. The uncertainty of whether the pricing of new antibiotics can recoup the cost of research and earn a profit also hinders the development of antibiotics [8]. A rapid, effective, and cost-saving alternative to new treatments is to find other drugs with antimicrobial potential [9].

5-fluorouracil (5-FU) was licensed for the treatment of cancer in 1962, and many subsequent studies have shown that 5-FU is also effective against certain Gram-positive and Gram-negative pathogens [10]. 5-FU is a chemical compound that resembles uracil by replacing hydrogen atoms with fluorine atoms at the C-5 position. It enters cells using the same transport mechanism as uracil. Its antibacterial and anticancer effects predominantly stem from active metabolites: fluorodeoxyuridine monophosphate (FdUMP), fluorodeoxyuridine triphosphate (FdUTP), and fluorouracil triphosphate (FUTP) [11]. While 5-FU and its derivatives have demonstrated clinical efficacy in various cancers, their potential for antibacterial applications remains underexplored. A previous antibacterial study of *Escherichia coli* (*E. coli*) revealed that 5-FU mainly inhibits the reductive methylation of deoxyuridine monophosphate (dUMP) by inhibiting thymidylate synthase [12]. DNA-synthesis cessation caused by deoxythymidine monophosphate (dTMP) starvation leads to thymine-less death. In another study, it was found that 5-FU can inhibit the synthesis of the cell-wall mucopeptide of *Staphylococcus aureus* (*S. aureus*) to a certain extent [13]. A subsequent study showed that exposure to 5-FU led to the perforation and rupture of the transparent membrane of *Pseudomonas aeruginosa* (*P. aeruginosa*), and wrinkling, shrinkage, and a loss of content were also observed [14]. 5-FU has also been shown to induce thymine-less death in Methicillin-resistant *Staphylococcus aureus* (MRSA) by inhibiting thymidine synthesis [15]. Bean et al. also observed a bacteriostatic effect of 5-FU against *Streptococcus pneumoniae* [16]. In addition, one study demonstrated the non-toxicity of 5-FU and its ability to inhibit the growth of *P. aeruginosa* biofilm, while another study obtained the same results on *Staphylococcus epidermidis* [17]. In another study, the resistance and mechanism of action of 5-FU in *Mycobacterium tuberculosis* (Mtb) were analyzed in detail [18]. However, the antimicrobial activity of the 5-FU against *S. suis* and the underlying mechanisms remain unclear. This study aimed to explore the possible antimicrobial potential of 5-FU against *S. suis* as an alternative to antibiotics.

## 2. Materials and Methods

### 2.1. Bacterial Strains and Growth Conditions

Information on the bacterial strains used in this study, including growth media and temperature, is displayed in Table 1. *Actinobacillus pleuropneumoniae* CVCC 265 (APP) [19]*, Escherichia coli* O157:H7 EDL933 (*E. coli*), *Glaesserella parasuis* SH0165 [20], *Salmonella Typhimurium* SAT52, and *S. suis* HA9801 were preserved in our laboratory. *Staphylococcus aureus* ATCC25923 and Group B *Streptococcus* ATCC12386 (GBS, *Streptococcus agalactiae*) were purchased from American Type Culture Collection (ATCC). Two strains (strain numbers SS-1 and SS-2) of *S. suis* were clinically isolated from the same pig farm in Luoyang, China. Samples such as feces and nasopharyngeal swabs were collected aseptically from the swine farm and inoculated on Blood agar plates, and incubated at 37 °C for 24 h. Then, suspicious colonies were selected from plates for Gram-staining, microscopic examination, and PCR identification. SS-1 and SS-2 have been identified as *S. suis* type 2 (Figure S3).

### 2.2. Construction of thyA Deletion Mutant Strain of S. suis

The construction of a *thyA* gene deletion strain of *S. suis* HA9801 was performed according to the procedure previously described [21]. The upstream and downstream regions of the *thyA* gene were amplified using *thyA*-A/*thyA*-B and *thyA*-C/*thyA*-D primers, respectively, by Polymerase Chain Reaction (PCR) (Table 2). After digestion with the corresponding restriction enzymes *Sal I* and *BamH I*, two DNA fragments were fused to a fragment AD without the target gene through fusion PCR. Subsequently, the fragment AD was cloned to a pSET4s vector. The recombinant plasmid was transformed into competent cells of *S. suis* by electroporation. After a two-step allelic exchange, the clones sensitive to spectinomycin were selected and the presence of *thyA* in the genome was detected by PCR using the specific primers listed in Table 2. The mutant was further validated through DNA sequencing.

### 2.3. Cytotoxicity Assay

The cell counting Kit-8 (CCK-8, Servicebio, Wuhan, China) colorimetric assay was used to measure the cytotoxicity of 5-FU with triplicate experiments for each set of conditions, according to the protocol of the manufacturer. Briefly, the HEP-2 cells were inoculated at a density of 5 × 10^4^ cells/mL into 96-well plates (DMEM containing 10% fetal bovine serum), and grown at 37 °C with different concentrations of 5-FU (0, 10, 5, 2.5, 1.25, and 0.625 µg/mL) for 24 h. Then, a CCK-8 solution of 10 µL was added to each well of the 96-well plate and the cells were incubated in the incubator for 1 h. The absorbance of each well was measured at 450 nm using a Synergy2 multi-function microplate reader (BioTek, Winooski, VT, USA). The toxicity of PK-15 cells was determined by the same method.

### 2.4. Resistance-Development Studies

To isolate spontaneous 5-FU^R^ mutants, cultures of WT SS were grown to the logarithmic phase (OD_600_ = 0.8) at 37 °C, and were diluted in fresh TSB medium containing 5-FU (0.625 µg/mL) at a ratio of 1:100 and incubated overnight. The MIC values were measured after 5 consecutive days of sub-culture. This step was performed repeatedly for 3–4 weeks. The 5-FU^R^ phenotype of colonies arising after 3–4 weeks’ incubation was confirmed. We tested the MIC of approved drugs on 5-FU^R^.

### 2.5. Determination of Minimum Inhibitory Concentration (MIC) and Minimum Bactericide Concentration (MBC)

The MIC and MBC were determined using the broth micro-dilution method based on the guidelines of the Clinical Laboratory Standards Institute (CLSI) [23]. *S. suis* HA9801 was cultured to the logarithmic growth phase, and the suspended culture medium was transferred as a 1:100 ratio to 96-well plates, and 5-FU at final concentrations of 0.625, 1.25, 2.5, 5, 10, 20, 40, 80, 160, and 320 µg/mL was added to different wells. The plates were subsequently incubated at 37 °C for 24 h. The MIC was determined as the lowest concentration of 5-FU at which no visible growth was observed in the wells upon visual inspection. Furthermore, bacterial suspensions from the wells used for MIC determination were spot-plated onto Tryptic Soy Agar (TSA) plates and cultured at 37 °C for 24 h. The MBC was determined as the lowest concentration of 5-FU at which no colony growth was observed on the TSA plates. The MIC and MBC of other strains were determined using the same method.

### 2.6. Bacterial Growth Curves

To evaluate the inhibitory influence of 5-FU on *S. suis*, the bacterial growth curve was delineated using the assaying OD_600_ nm values and viable bacterial counts of the culture medium at different time points. *S. suis* in the logarithmic growth phase (10^6^ CFU/mL, 100 µL) was inoculated into 10 mL of sterile TSB medium, and 5-FU with concentrations of 0, 2.5, 1.25, and 0.625 µg/mL were added to the bacterial suspension, respectively. Then, the culture broths were incubated at 37 °C, 180 rpm/min, for 12 h. The turbidity was monitored by measuring the OD_600_ nm for 0, 2, 4, 6, 8, 10, and 12 h, and the bactericidal curve was drawn. Colony counts of *S. suis* were performed according to methods in a previous study [24], and 100 μL of bacterial solution was uniformly coated on a TSA plate every 2 h and incubated at 37 °C for 24 h. Growth curve of *S. suis* was plotted using plate counting method.

### 2.7. Colony-Forming Unit Assay

Overnight growth of *S. suis* cultures were diluted in a fresh medium at a ratio of 1:100 and cultured to the mid-exponential phase. Each culture was then diluted in a ratio of 1:10 into a fresh medium and treated separately with different antimicrobials. In total, 100 μL culture solution per hour was taken and spread on the plate. Then, it was incubated overnight at 37 °C in the absence of antimicrobials. Colony-forming units were counted the next day.

### 2.8. Scanning Electron Microscope (SEM)

Scanning electron microscope (SEM) analysis was performed to characterize the effect of 5-FU on the morphology and structure of *S. suis* [25]. In brief, *S. suis* WT and Δ*thyA* were treated with different concentrations of 5-FU (0, 2.5, 1.25, and 0.625 µg/mL) and incubated for 12 h at 180 r/min and 37 °C. All cultures were centrifuged at 8000× *g* for 10 min and then washed twice with PBS. The bacterial cells were then fixed with 2.5% glutaraldehyde for 2 h and dehydrated with gradient alcohols (20%, 30%, 50%, 60%, 70%, 80%, 90%, and 100%) for 20 min at room temperature. Finally, the bacterial cell surfaces were sprayed with gold using a sputter coater (8 mA, 4 min), and the samples were examined by using the SEM (Jeol, Tokyo, Japan), and the image magnification was set at 5000×.

### 2.9. Transmission Electron Microscope (TEM)

Transmission electron microscope (TEM) observation was employed to analyze the effect of 5-FU on the morphology of *S. suis* cells. The experimental procedures were performed as described previously with some modifications [26]. The cultured *S. suis* WT and Δ*thyA* suspensions added with different concentrations of 5-FU (0, 2.5, 1.25, and 0.625 µg/mL) were washed twice with sterile PBS, and fixed in a 2.5% glutaraldehyde solution overnight. Subsequently, the cells were treated with 2% osmium tetroxide in the dark for 2 h, followed by dehydration with gradient ethanol. Finally, *S. suis* cells were implanted in an epoxy resin, observed using the TEM (Hitachi, Tokyo, Japan), and photographed.

### 2.10. Lactate Dehydrogenase (LDH) Assay

The release of LDH from bacterial cells to the culture medium was detected using the lactate dehydrogenase-activity-detection kit (Beijing Solarbio Science & Technology Co., Ltd., Beijing, China) to evaluate the membrane damage [27]. An *S. suis* suspension with different concentrations of 5-FU (0, 2.5, 1.25, and 0.625 µg/mL) was cultured at 37 °C for 8 h. Bacterial cultures were centrifuged at 8000 rpm/min for 5 min, and then the supernatant was mixed with reagents according to the instructions. The LDH release was quantified by UV absorbance with measurements at 450 nm.

### 2.11. Determination of Genomic DNA Integrity

The effect of 5-FU on the genomic DNA of *S. suis* WT and Δ*thyA* was evaluated by agarose gel electrophoresis [28]. Bacterial suspensions of *S. suis* WT and Δ*thyA* were prepared as described above and mixed with different concentrations of 5-FU (0, 2.5, 1.25, and 0.625 µg/mL) for 30 min. Following treatment, DNA was extracted by lysing *S*. *suis* WT and Δ*thyA* using lysozyme and proteinase K. Bacterial lysates were collected and processed using the M5 HiPer Bacterial Genomic DNA Kit (Mei5bio, Beijing, China). The integrity of the DNA samples was observed using 1% agarose gel electrophoresis at 120 V for 30 min. Finally, the gels were photographed using the Tanon 1600 gel-imaging system (Tanon, Shanghai, China).

### 2.12. RNA Extraction and Quantitative Reverse Transcription Polymerase Chain Reaction (qRT-PCR)

The total RNAs of *S. suis* were extracted according to the previously described procedure [29]. *S. suis* WT and Δ*thyA* were incubated with different concentrations of 5-FU (0, 2.5, 1.25, and 0.625 µg/mL) to the logarithmic growth stage. To collect the bacteria, all bacterial cultures were centrifuged at 8000× *g* for 5 min. The bacterial total RNA was extracted using the TRIzon reagent (Kangwei Biotech, Beijing, China). Afterward, reverse transcription was performed using the M5 HiPer one-step RT-PCR Kit (Mei5bio, Beijing, China). The cDNA was preserved at −20 °C. The 2× M5 HiPer SYBR Premix EsTaq (Mei5bio, Beijing, China) was used for quantitative fluorescence PCR detection. Gene-specific forward and reverse primers were designed using Primer5 software (Premier Biosoft International) (Table 2). The 16S rRNA gene was used as an internal control gene. The PCR reaction conditions were the following: initial denaturation at 95 °C for 30 s; denaturation at 95 °C for 10 s, annealed at 60 °C for 30~60 s, 40 cycles. The relative expression genes were analyzed by using the 2^−∆∆Ct^ method [24].

### 2.13. Animal Experiments

The animal experiments were approved by the Animal Care and Use Committee of Henan University of Science and Technology (approval number: SKKUIACUC-20-04-14-3). We used a total of 18 female BALB/c mice (4 weeks old) and randomly divided into six groups. All groups of BALB/c mice (3 mice per group) were inoculated with an intraperitoneal injection of bacteria inoculum and administered medication through tail-vein injection for treatment. The groups are as follows: Group 1: WT + solvent (PBS) group; Group 2: WT + 20 μg/g 5-FU; Group 3: Δ*thyA* + solvent; Group 4: Δ*thyA* + 20 μg/g 5-FU; Group 5: WT + 20 μg/g 5-FU + T; and Group 6: WT + 20 μg/g AMX.

To evaluate the effect of 5-FU on the colonization ability of WT and Δ*thyA* in mice, we diluted the cultured bacterial solution with PBS to 5 × 10^6^ CFU/mL. Group 1, Group 2, Group 5, and Group 6 were inoculated with 200 µL of WT intraperitoneal, while Groups 3 and 4 were intraperitoneally injected with 200 µL of Δ*thyA*. Treatment was then administered with different concentrations of 5-FU or AMX (0, 20 μg/g) through the tail vein at 2 h, 14 h, 26 h, and 38 h after infection. After treatment, all mice were euthanized, and the lungs, livers, and spleens were taken out and sterilized for bacterial counts and histopathological observation. At the end of the experiment, dilutions of the homogenates were plated on TSA plates to determine the bacterial loads.

### 2.14. Statistical Analysis

A one-way ANOVA was used for determining statistical significance and was calculated using GraphPad Prism 8.0. A *p*-value of ≤0.05 was considered statistically significant.

## 3. Results

### 3.1. 5-FU Exhibits Effective Antibacterial Activity and Safety In Vitro

**Figure 1:**
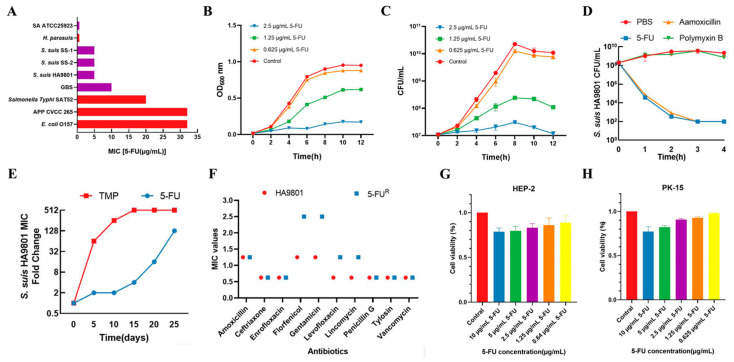
5-FU has significant antibacterial and bactericidal effects, a low frequency of drug resistance, and low cytotoxicity. (**A**) The MIC of 5-FU against Gram-negative (red) and Gram-positive (purple) bacteria. See also Table 1. (**B**,**C**) The bacterial-growth curve of *S. suis* HA9801 after treatment with 5-FU. The bacterial-growth curves were measured by the optical density at 600 nm and colony-forming units every 2 h following inoculation. (**D**) Colony-forming units (CFUs mL^−1^) after a 4 h treatment of *S. suis* HA9801 with PBS (solvent control), 20 µg/mL 5-FU (4 × MIC), 2.5 µg/mL amoxicillin (4 × MIC), or 2 mg/mL Polymyxin B (4 × MIC). The data points of 1 × 10^2^ CFU mL^−1^ were below the detected level. The colony-forming units of two clinically isolated antibiotic-resistant strains SS-1 and SS-2 of *S. suis* are shown in Figure S1. Each datum represents three biological replicates. The mean ± SD is shown. (**E**,**F**) The resistance to multiple changes of *S. suis* HA9801 to 5-FU and TMP after 30 consecutive passages of each drug, and the cross-resistance of 5-FU^R^ to commonly used clinical antimicrobials. (**G**,**H**) Cytotoxicity of 5-FU at different concentrations on HEP-2 and PK-15 cells.

To examine the antibacterial activity of 5-FU, the MIC and MBC were determined for *S. suis*. The results revealed that 5-FU displayed strong activity in comparison to commonly employed antibiotics for the treatment of *S. suis*. The MIC of 5-FU against the *S. suis* HA9801 strain was 5 µg/mL and the MBC was 10 µg/mL (Figure 1A). Notably, 5-FU exhibited equivalent bactericidal activity against SS-resistant strains when compared to the antibiotics typically used against *S. suis* (Table 1). Additionally, growth-curve analysis demonstrated that 5-FU effectively inhibited the growth of *S. suis* HA9801 promptly and efficiently (Figure 1B,C). At the same time, 5-FU showed similar bactericidal activity against *S. suis* HA9801, as well as drug-resistant strains SS-1 and SS-2 (Figure 1D; Figure S1). Furthermore, to evaluate the spectrum of bacterial sensitivity to 5-FU, the MIC and MBC were measured against several clinically relevant pathogens. The results indicated that 5-FU significantly eradicated various Gram-negative and Gram-positive pathogens, including *S. aureus*, *S. agalactis*, *E. coli*, and *Salmonella typhimurium* (*S. typhimurium*) (Figure 1A and Table 1).

To further characterize its potential as an antimicrobial, we determined the frequency of bacterial resistance toward 5-FU. *S. suis* HA9801 was subjected to continuous sub-culturing under sub-MIC conditions of 5-FU for a period of 30 days, resulting in the acquisition of a mutant strain designated as 5-FU^R^, which exhibited resistance to 5-FU. In vitro resistance-induction experiments demonstrated a remarkably sluggish development of resistance towards 5-FU (Figure 1E), and MIC results for 5-FU^R^ revealed no cross-resistance with other commonly utilized clinical antimicrobials (Figure 1F). Furthermore, we assessed its cytotoxicity on mammalian cells. Both HEP-2 cells and PK-15 cells were employed to evaluate the cytotoxic effects induced by 5-FU. Our findings demonstrated that 5-FU did not elicit cytotoxic effects on mammalian cells at concentrations of 10 µg/mL (Figure 1G,H). In summary, these results affirm that 5-FU has good potential for substitute resistance, yet its bactericidal mechanism is not clear.

### 3.2. 5-FU Disrupts the Cell Wall and Bacterial Membrane Morphology and Induces DNA Damage in S. suis

**Figure 2:**
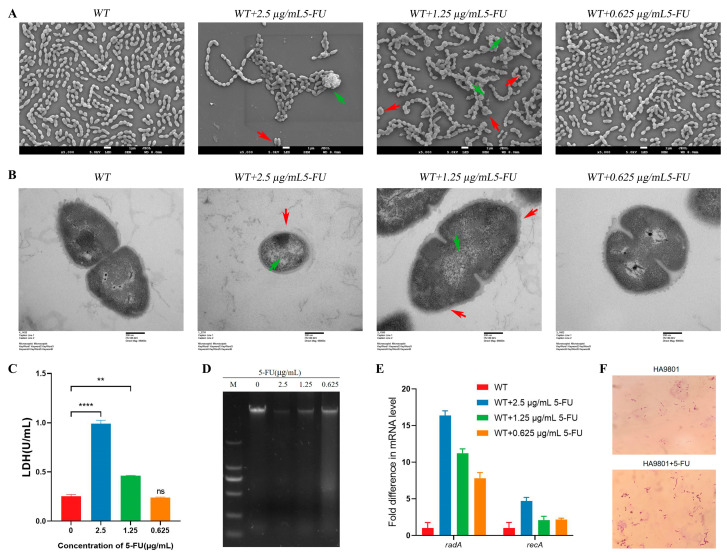
5-FU disrupts the cell wall and bacterial-membrane morphology and induces DNA damage in *S. suis*. (**A**) SEM images of *S. suis* treated with different concentrations of 5-FU. The red arrow indicates swelling and deformation of the bacterial membrane, while the green arrow indicates the bacterial fragments. (**B**) TEM images of *S. suis* treated with different concentrations of 5-FU. The green arrow indicates that the bacterial chromatin is not obvious, while the red arrow indicates that the bacterial capsule layer was dissolved, the cell membrane structure of *S. suis* ruptured, and the cellular contents leaked. (**C**) LDH release of *S. suis* under different concentrations of 5-FU treatment. Data are shown as the means ± SDs. ** *p* < 0.01, **** *p* < 0.0001, ^ns^ *p* < 0.05. (**D**,**E**) DNA electrophoretogram and expression levels of *recA* and *radA* genes in *S. suis* under different concentrations of 5-FU treatment. (**F**) Light-microscopic observation of *S. suis* treated or untreated with 5-FU.

Surprisingly, when observing *S. suis* treated with 5-FU under a light microscope, A notable enlargement of the cells was observed (Figure 2F). To obtain clues about the potential bactericidal effect of 5-FU, scanning electron microscopy (SEM) was used to visualize the morphological changes in *S. suis* cells upon treatment with 5-FU. The SEM results revealed that *S. suis* cells treated with 0.625 µg/mL of 5-FU displayed morphological changes comparable to those of untreated *S. suis*, with a full morphology, smooth surface, and without obvious damage. However, bacterial cells treated with 1.25 µg/mL of 5-FU exhibited partial cell rupture, as well as cell swelling and deformation, while those cells incubated with 2.5 µg/mL of 5-FU displayed increased bulging, accompanied by a higher presence of bacterial debris. Furthermore, the number of cells within the observed field of view decreased with increasing doses of 5-FU, indicating higher concentrations resulting in greater cell death (Figure 2A). These findings suggested that 5-FU can affect the morphology of *S. suis* in a concentration-dependent manner, resulting in bacterial death. To further elucidate the contributing factors, transmission electron microscopy (TEM) was used to examine the cell membrane and cell wall structure of *S. suis*. TEM analysis revealed that untreated *S. suis* possessed an intact cell structure, characterized by a clear cell membrane, evident cytoplasm and nuclear areas, and intracellular dark regions representing cellular contents. Conversely, treated groups exhibited various degrees of cellular damage. Specifically, cells treated with 1.25 µg/mL of 5-FU displayed inconspicuous chromatins, a thinning of the capsule layer, and dim and fragmentary cell membranes. After treatment with a concentration of 2.5 µg/mL of 5-FU, the morphology of certain cells exhibited irregularities, the bacterial capsular layer underwent dissolution, the cell membrane structure of *S. suis* ruptured, and the leakage of cellular contents occurred (Figure 2B). Subsequently, to further investigate the impact of 5-FU on the cell membrane of *S. suis*, an LDH assay was performed to measure the release of cytoplasmic enzymes from bacterial cells into the culture medium, serving as an indicator of damaged cell membranes. A concentration-dependent release of LDH was observed when bacterial cells were exposed to 5-FU. Specifically, bacterial cells exposed to 0.625 µg/mL of 5-FU demonstrated minimal LDH release, which did not significantly differ from the untreated group. However, LDH release increased significantly when bacterial cells were exposed to solutions containing 1.25 and 2.5 µg/mL of 5-FU (Figure 2C). These results suggest that 5-FU induces cell-wall damage and alterations in the cell-membrane permeability of *S. suis* in a dose-dependent manner, and promotes the entry of 5-FU into the bacteria.

According to reports, 5-FU is mainly used to induce bacterial thymine-less death by inhibiting thymidine synthesis, which subsequently leads to significant DNA damage [30]. To examine the hypothesis that 5-FU treatment induces DNA damage in bacteria, agarose gel electrophoresis was employed to analyze the damage of 5-FU on the genomic DNA of *S. suis*. The DNA electrophoretogram of the control samples displayed clear and bright DNA bands, whereas the DNA bands darkened or disappeared after treatment with 5-FU. Notably, the intensity of the DNA bands darkened with increasing concentrations of 5-FU. Remarkably, the genomic DNA bands of *S. suis* nearly vanished when the cells were treated with 2.5 µg/mL of 5-FU (Figure 2D). To assess whether the incorporation of 5-FU into DNA would trigger a genetic toxic stress response, we investigated the impact of 5-FU treatment on the expression of the *recA* and *radA* genes (associated with DNA damage) of *S. suis* by real-time PCR [31]. The gene-expression levels of *recA* and *radA* in the 5-FU-treated group were significantly higher than compared to the untreated group (Figure 2E). These outcomes provide compelling evidence that the incorporation of 5-FU can indeed induce DNA damage in *S. suis*.

### 3.3. 5-FU Causes Damage to S. suis by Inhibiting Thymidine Synthesis

**Figure 3:**
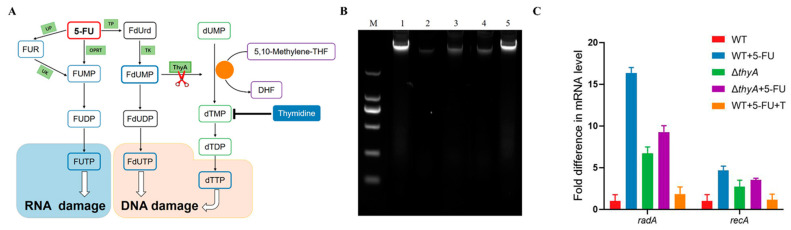
Antibacterial mechanisms of 5-FU in *S. suis* HA9801. (**A**) A partial representation of 5-FU metabolic pathway. 5-FU uses the same metabolic mechanisms as uracil to enter the cell, metabolizing into FdUMP, FdUTP, and FUTP to exert antibacterial effects. (**B**) DNA electrophoretogram in *S. suis* WT and *S. suis* Δ*thyA* treated or untreated with 5-FU. M, marker; 1, WT; 2, WT + 5-FU; 3, Δ*thyA*; 4, Δ*thyA* + 5-FU; 5, WT + 5-FU + T. (**C**) Expression levels of *recA* and *radA* genes in *S. suis* WT and *S. suis* Δ*thyA* treated or untreated with 5-FU.

5-FU is an inhibitor of thymidine synthase. To explore the potential thymine depletion caused by 5-FU in *S. suis*, we also investigated the MIC and MBC of 5-FU against *S. suis* HA9801 in the presence of exogenous thymidine, using the same procedure as for the compound alone. As a negative control, amoxicillin (AMX) was employed. As expected, the MIC value of AMX remained unaffected by the addition of an exogenous thymidine, while the MIC value of 5-FU was strongly increased by the addition of thymidine. (Figure 3A). This suggests that the exogenous addition of thymidine rescues the killing effect of 5-FU on *S. suis*, prompting us to analyze the possible bactericidal pathway of 5-FU in *S. suis* as causing thymidine-less death by inhibiting thymidine synthesis. Consequently, we constructed *S. suis* HA9801 Δ*thyA* (Figure S2) to further validate the bactericidal mechanism of 5-FU against *S. suis*. The deletion of the *thyA* gene in *S. suis* HA9801 resulted in the inability of the bacteria to survive in the absence of thymidine. We subsequently determined the MIC and MBC of 5-FU against Δ*thyA*, and the results showed that the MIC was 80 µg/mL and the MBC was 320 µg/mL (Table 1). These findings indicate a statistically non-significant inhibitory and bactericidal effect of 5-FU in Δ*thyA* compared to the wild strain. Thus, we can tentatively conclude that 5-FU primarily induces thymine-less death in *S. suis* by impeding thymidine synthesis.

To determine whether the bactericidal effect of 5-FU on *S. suis* was caused by targeted thymidine synthesis, we used Δ*thyA* to carry out a DNA-damage test. The analysis of DNA gel imaging revealed significantly darker band intensities in both the Δ*thyA* group and the Δ*thyA* + 5-FU group, while the WT + 5-FU + T group exhibited brighter bands (Figure 3B). Similarly, the expression levels of the DNA-damage-response genes *recA* and *radA* were significantly higher in both the Δ*thyA* group and the Δ*thyA* + 5-FU group compared to the control group, but there was no significant difference between the WT + 5FU + T group and the control group (Figure 3C). These results suggest that *thyA* serves as a crucial target for 5-FU-induced DNA damage in *S. suis* because *S. suis* Δ*thyA* shows similar results to *S. suis* WT treated with 5-FU, and exogenous thymidine can mitigate the damage caused by 5-FU in *S. suis* WT but fails to do so in *S. suis* Δ*thyA*.

### 3.4. 5-FU Reduces S. suis Colonization of Tissues in Mice

**Figure 4:**
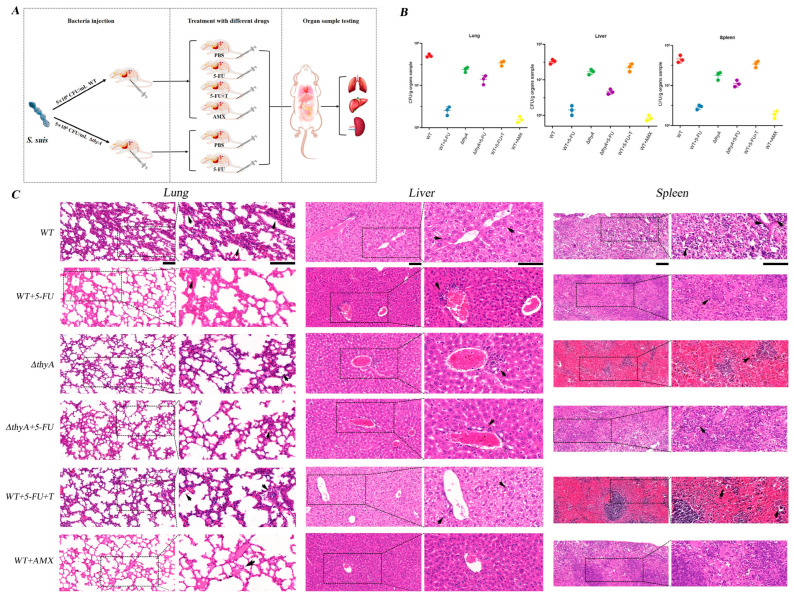
Antibacterial experiment in mice. (**A**) Schematic diagram of mouse infection model. (**B**) Bacterial load in the lung, liver, and spleen of *S. suis* WT-infected or *S. suis* Δ*thyA*-infected BALB/c mice treated with 5-FU (20 µg/g) and AMX (2 mg/g). (**C**) Histopathologic changes of *S. suis* infections caused (black scale bar = 50 µm, magnification = 200×). The black arrows in figure showed the histopathological changes in the lung, liver, and spleen.

Given the favorable bactericidal efficacy and low-dose cytotoxicity of 5-FU, we proceeded to investigate its effects in mice. We inoculated 5 × 10^6^ CFU/mice with WT or Δ*thyA* in BALB/c mice. The experimental procedures are outlined in Figure 4A, and the bacterial load in tissues and organs, as well as histopathological sections, were evaluated two days post-infection. The results demonstrated a significant reduction in bacterial load in each organ of the 5-FU treatment group, akin to that observed with the clinical drug amoxicillin when compared to the control group. However, the addition of exogenous thymidine substantially diminished the therapeutic effect of 5-FU. Moreover, no notable changes in bacterial load were observed in all organs of the Δ*thyA* group, irrespective of treatment with or without 5-FU (Figure 4B). The H&E staining section showed loss of lung tissue structure and alveolar congestion in the control group; large vesicles in the cytoplasm of hepatocytes and inflammatory infiltration of the portal vein; and moderate splenic congestion. However, there was obvious recovery after 5-FU treatment, and only mild lesions were found, which was similar to that of amoxicillin. Similarly, different degrees of pathological changes were seen in the Δ*thyA* group with or without 5-FU treatment (Figure 4C), which was consistent with the results of in vitro experiments.

## 4. Discussion

*Streptococcus suis* (*S. suis*) is a zoonotic pathogen widely distributed around the world, which not only causes huge economic losses to the swine industry, but also threatens human lives. The increasing resistance to conventional antibiotics underscores the urgent need to explore novel and effective alternatives. A rapid, effective, and cost-saving alternative to new treatments is to find other drugs with antimicrobial potential [9]. In this context, we examined the efficacy of 5-fluorouracil (5-FU), an FDA-approved drug, in suppressing and eradicating *S. suis*. 5-FU was licensed for the treatment of cancer in 1962 [10], and many subsequent studies have shown that 5-FU is also effective against a variety of Gram-positive and Gram-negative bacteria [12,13].

In this study, we first measured the antibacterial activity of 5-FU against *S. suis*. Remarkably, when compared to the commonly used antibiotic amoxicillin, 5-FU demonstrated substantial bactericidal and bacteriostatic effects against *S. suis* HA9801, as well as multi-drug resistant strains SS-1 and SS-2 (Figure 1A–D; Figure S1). In addition, 5-FU exhibits effective antibacterial and bactericidal activity against many other clinically relevant bacteria, including *S. agalactis*, *S. aureus*, *Escherichia coli*, and *S. typhimurium* (Figure 1A and Table 1). Importantly, 5-FU has no cross-resistance to approved antimicrobials (Figure 1E,F), and cytotoxicity tests on HEP-2 cells and PK-15 cells showed the very weak cytotoxicity of 5-FU (Figure 1G,H). These results indicated that 5-FU is a promising alternative to antibiotics. We proceeded to observe structural damage to the cell wall and membrane of *S. suis* following treatment with 5-FU by SEM and TEM (Figure 2A,B). This observation was further substantiated by LDH assays, which confirmed the occurrence of cellular damage (Figure 2C). These findings align with previous studies conducted on *P. aeruginosa* [14], where exposure to 5-FU led to the perforation and rupture of the transparent membrane, accompanied by wrinkling, shrinkage, and a loss of content. Moreover, Chihiro et al. demonstrated that 5-FU induced thymine-less death in MRSA through the inhibition of thymidine synthesis [15], with thymine starvation leading to severe DNA damage [30]. Our DNA-damage test results for *S. suis* corroborated these findings, where 5-FU incorporation caused DNA damage in *S. suis* (Figure 2D,E).

The toxicity mechanism of 5-FU involves the incorporation of its metabolite FUTP into RNA molecules, which disrupts normal RNA processing and function [32]. Another major mechanism of 5-FU is the inhibition of thymidine synthesis. TS catalyzes dUMP metabolism to dTMP, which provides the only de novo source of thymidylate. Thymidylate is a necessary component for DNA replication and repair. 5-FU’s metabolite FdUMP irreversibly forms a ternary complex with TS and the methyl-group donor 5,10-CH2-THF [10,11]. This complex prevents the binding of the normal substrate, dUMP, inhibiting the synthesis of dTMP [33]. The inhibition of TS leads to downstream effects, such as the depletion of dTTP and cell thymine-less death [10,11]. Consequently, an accumulation of uracil nucleotides, including dUTP, may occur [34]. Imbalances in the deoxynucleotide pool can severely disrupt DNA synthesis and repair, resulting in lethal DNA damage [30]. Studies have demonstrated that TS is an important factor contributing to the rapid accumulation of dUMP in cells or bacteria, and the chain reaction triggered is an increase in dUMP at the cellular level [11]. Furthermore, 5-FU of fluorine is synthesized from FdUTP into DNA as metabolism proceeds. This consequence triggers cellular and bacterial DNA strand damage or even breaks, which triggers cellular and bacterial damage and death [30].

Chihiro et al. established that 5-FU induces thymine-less death in *S. aureus* through the exogenous addition of pyrimidine nucleosides [15]. In this study, we verified the antibacterial mechanism of 5-FU by adding thymidine exogenously. As expected, the addition of thymidine substantially reduced the antibacterial efficacy of 5-FU (Table 1), confirming its mode of bactericidal action as inducing thymine-less death in *S. suis*.

Then, we constructed a mutant with a deletion of the thymidine synthase gene *thyA* (Figure S2). Our assessment of 5-FU’s antibacterial activity against Δ*thyA* (Table 1) and DNA damage tests (Figure 3B,C) indicated that *thyA* represents the primary target of 5-FU’s bactericidal action, whereby the inhibition of thymidine synthesis leads to thymine-less death in *S. suis*. Since the antibacterial activity of 5-FU against Δ*thyA* is not entirely abolished, this suggests the existence of additional targets for 5-FU, which explains the reason for the damage to the cell membrane of bacteria treated with 5-FU.

Encouraged by the significant in vitro antimicrobial activity, the lack of cross-resistance to approved antimicrobials, and the low toxicity of 5-FU, we proceeded to evaluate its efficacy in an *S. suis*-infected mouse model (Figure 4A). The results demonstrated a significant reduction in bacterial load and decreased tissue and organ damage in mice treated with 5-FU compared to the control group. Importantly, the therapeutic effect was comparable to that of the control group using the antibiotic amoxicillin (Figure 4B,C). However, for *S. suis* Δ*thyA*, the therapeutic effect of 5-FU is not significant, which further proves that *thyA* is a crucial target for 5-FU sterilization.

## 5. Conclusions

In summary, our study demonstrated the antibacterial activity of 5-FU against *S. suis*, along with its lack of cross-resistance to approved antimicrobials, suggesting its potential as a new class of alternative drugs. The primary mechanisms underlying its antimicrobial effects involve thymine-less death induction and membrane damage. These findings pave the way for the development of novel antibacterial drugs targeting *S. suis* infections based on the properties of 5-FU. However, more studies are needed, including therapeutic experiments on pigs and experiments to assess its clinical therapeutic potential.

## Figures and Tables

**Table 1:** Strains used and the MICs and MBCs of 5-FU or AMX against Gram-negative and Gram-positive bacteria.

Isolate	Strain/ Subspecies	MIC 5-FU (µg/mL)	MBC 5-FU (µg/mL)	MIC AMX (µg/mL)	Temperature (°C)	Media ^a^
*Actinobacillus pleuropneumoniae*	CVCC 265	32	>128		37	TSB Broth + NAD
*Escherichia coli*	O157:H7	32	>128		37	LB Broth
*Glaesserella parasuis*	[20]	0.625	5		37	TSB Broth + serum + NAD
GBS	[19]	10	40		37	TSB Broth
*Salmonella Typhimurium*	SAT52	20	40		37	LB Broth
*Staphylococcus aureus*	ATCC25923	0.625	10		37	TSB Broth
*Streptococcus suis*	HA9801	5	10	1.25	37	TSB Broth
*Streptococcus suis*	HA9801	40	160	1.25	37	TSB Broth + Thymine
*Streptococcus suis*	HA9801 Δ*thyA*	80	320	1.25	37	TSB Broth + Thymine
*Streptococcus suis*	SS-1	5	10	2.5	37	TSB Broth
*Streptococcus suis*	SS-2	5	10	2.5	37	TSB Broth
*Streptococcus suis*	SS-1	20	80	2.5	37	TSB Broth + Thymine
*Streptococcus suis*	SS-2	40	160	2.5	37	TSB Broth + Thymine

^a^ TSB, Tryptone soya broth. LB, Luria Bertani. NAD, Nicotinamide adenine dinucleotide.

**Table 2:** Plasmids and primers used in this study.

Plasmids and Primers	Relevant Characteristics ^a^ or Sequence (5′-3′) ^b^	Size (bp)	References or Target Gene
Plasmids			
pSET4s	Thermosensitive suicide vector; Spc^R^		[22]
pSET4s-Δ*thyA*	Knockout vector for *thyA* deletion; Spc^R^		This study
Primers			
*gdh*-F	GTTGAGCCTGAGCGTATCATC	425	An internal region of *gdh*
*gdh*-R	CCAGTCAAGACACCTGCATC		
*cps2*-F	ATTGGTAGGCACTGTCGTTGGTC	191	An internal region of *cps2*
*cps2*-R	AGAACTTAGCATTGTTGCGGTGG		
*thyA*-A	CCGCGTCGACCATCACTCGTCTTGAAATAATCGTT	792	The left arm of *thyA*
*thyA*-B	GGTCTTAGTATAGCAAATTCTAGCA		
*thyA*-C	TGCTAGAATTTGCTATACTAAGACCCTGGATTAGCAGTGAAGAACTTCGT	760	The right arm of *thyA*
*thyA*-D	ACGCGGATCCCATACGGGTTTTTCTCGTCTTTTGG		
*thyA*-ORF-S	GTCTAAGGGCGAGTTTCC	554	An internal region of *thyA*
*thyA*-ORF-A	GACGGAGTAATTCTTCAGC		
*thyA*-XY-S	AGTCCTCAATCCTGCCTACATCGTT	1636	A fragment containing *thyA*
*thyA*-XY-A	TCGCCATTAAATTGACC		
Q-16S rRNA-F	GTTGCGAACGGGTGAGTAA		An internal region of 16S rRNA
Q-16S rRNA-R	TCTCAGGTCGGCTATGTATC G		
Q-*recA*-F	TCCCCTGAGTCTGGCTGTG		An internal region of *recA*
Q-*recA*-R	ATGGTGGTATTGCTGCCTTTA		
Q-*radA*-F	AACATAGTCGGCGTCACCA		An internal region of *radA*
Q-*radA*-R	AAAATCGCTTCGGCTCCAC		

^a^ Spc^R^, spectinomycin resistant. ^b^ Underlined nucleotides represent restriction sites.

## Data Availability

Data will be made available on request.

## Supplementary Materials

The following supporting information can be downloaded at https://www.mdpi.com/article/10.3390/ani14091286/s1, Figure S1: Colony-forming units (CFUs mL^−1^) after 4 h treatment of SS-1 and/or SS-2 with different drugs; Figure S2: PCR confirmation of the knockout mutant strain Δ*thyA*; Figure S3: Identification of strain types and serotypes of SS-1 and SS-2.
