# Supplementary material for: Tackling Antibiotic Resistance: Exploring 5-Fluorouracil as a Promising Antimicrobial Strategy for the Treatment of Streptococcus suis Infection
Source: Animals (Basel). 2024 Apr 24;14(9):1286. doi: 10.3390/ani14091286 (PMC11083182; doi:10.3390/ani14091286)
Supplement: Supplementary file 1 [file animals-14-01286-s001.zip › animals-2941341-supplementary.pdf]

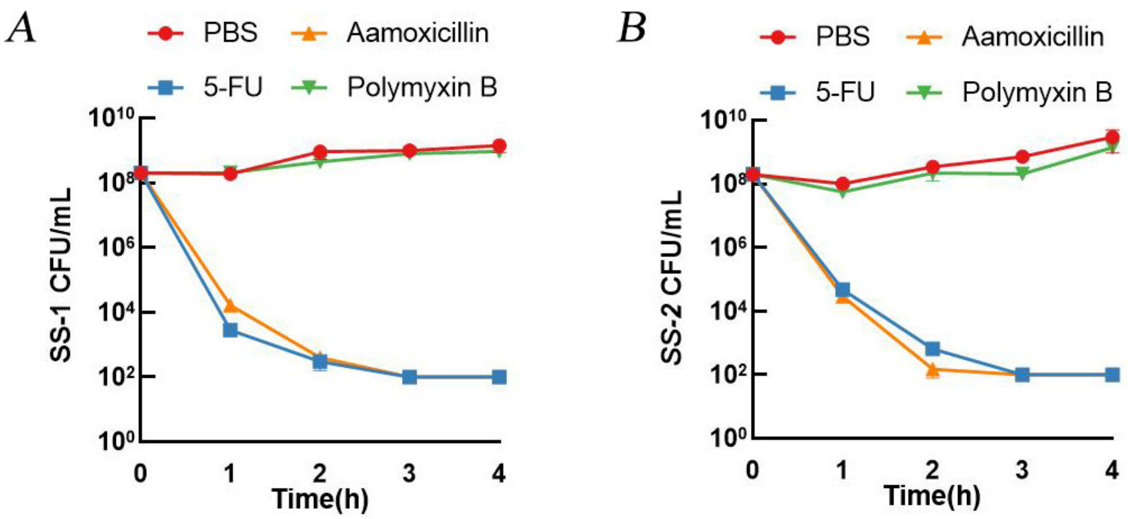

Figure S1, related to Fig 1D. Colony forming units (CFUs mL^{-1}) after 4-h treatment of SS-1 and/or SS-2 with different drugs. (A) Colony forming units (CFUs mL^{-1}) after 4-h treatment of SS-1 with PBS (solvent control), 20 µg/mL 5-FU (4×MIC), 10 µg/mL Amoxicillin (4×MIC), or 2 mg/mL Polymyxin B (4×MIC). **(B)** Colony forming units (CFUs mL^{-1}) after 4-h treatment of SS-2 with PBS (solvent control), 20 µg/mL 5-FU (4×MIC), 10 µg/mL Amoxicillin (4×MIC), or 2 mg/mL Polymyxin B (4×MIC). Data points at 1×10^2 CFU mL^{-1} are below the level of detection. Each data point represents 3 biological replicates. Mean ± SD are shown.

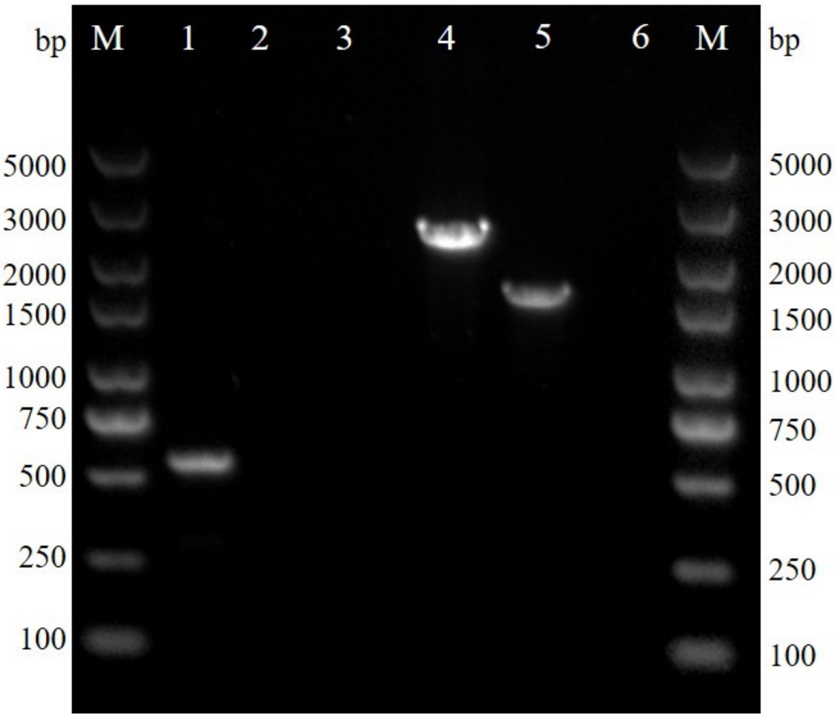

Figure S2. **PCR confirmation of the knockout mutant strain Δ*thyA*.** Lane 1, primers ORF-S/ORF-A, HA9801; lane 2, primers ORF-S/ORF-A, Δ*thyA*; lane 4, primers XY-S/XY-A, HA9801; lane 5, primers XY-S/XY-A, Δ*thyA*. Lanes 3 and 6 represent the negative control.

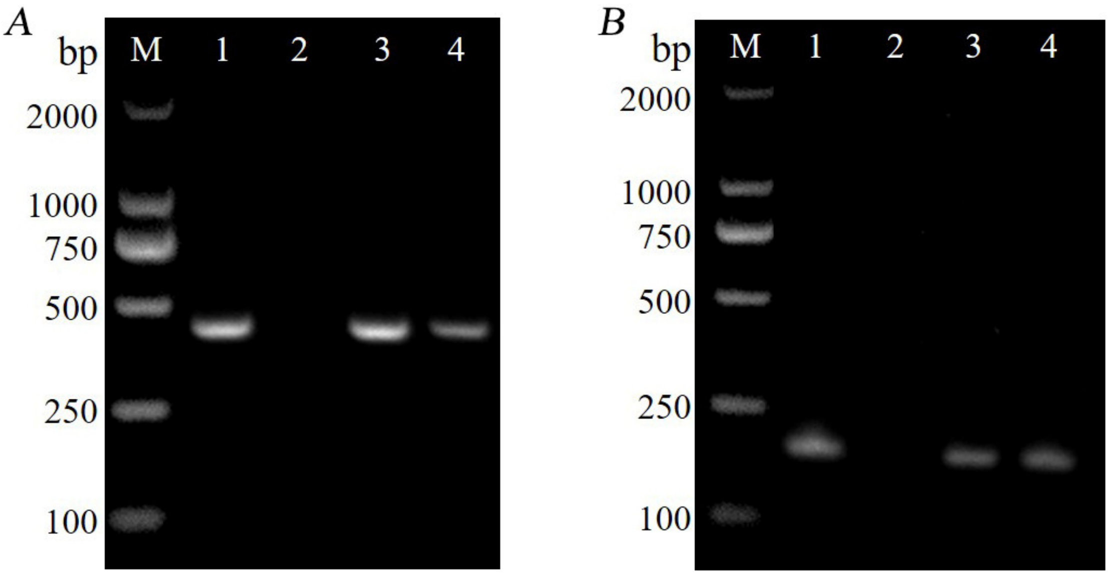

Figure S3. Identification of strain types and serotypes of SS-1 and SS-2. (A) PCR confirmation of *gdh* gene. Lane 1, HA9801; lane 2, negative control; lane 3, SS-1; lane 4, SS-2. **(B)** PCR confirmation of *cps2* gene. Lane 1, HA9801; lane 2, negative control; lane 3, SS-1; lane 4, SS-2.
